# Monitoring Results in Routine Immunization: Development of Routine Immunization Dashboard in Selected African Countries in the Context of the Polio Eradication Endgame Strategic Plan

**DOI:** 10.1093/infdis/jiw635

**Published:** 2017-07-01

**Authors:** Alain Poy, Maya M. V. X. van den Ent, Stephen Sosler, Alan R. Hinman, Sidney Brown, Samir Sodha, Daniel C. Ehlman, Aaron S. Wallace, Richard Mihigo

**Affiliations:** 1 World Health Organization, Regional Office for Africa, Brazzaville, Congo;; 2 United Nations Children’s Fund, New York, New York;; 3 GAVI, The Vaccine Alliance, Geneva, Switzerland;; 4 Task Force for Global Health, Decatur, Georgia;; 5 Bill and Melinda Gates Foundation, Seattle, Washington;; 6 United States Centers for Disease Control and Prevention, Atlanta, Georgia.

**Keywords:** Monitoring, Routine immunization, dashboard, endgame strategy, system strengthening, Polio.

## Abstract

**Background.:**

To monitor immunization-system strengthening in the Polio Eradication Endgame Strategic Plan 2013–2018 (PEESP), the Global Polio Eradication Initiative identified 1 indicator: 10% annual improvement in third dose of diphtheria- tetanus-pertussis–containing vaccine (DTP3) coverage in polio high-risk districts of 10 polio focus countries.

**Methods.:**

A multiagency team, including staff from the African Region, developed a comprehensive list of outcome and process indicators measuring various aspects of the performance of an immunization system.

**Results.:**

The development and implementation of the dashboard to assess immunization system performance allowed national program managers to monitor the key immunization indicators and stratify by high-risk and non–high-risk districts.

**Discussion.:**

Although only a single outcome indicator goal (at least 10% annual increase in DTP3 coverage achieved in 80% of high-risk districts) initially existed in the endgame strategy, we successfully added additional outcome indicators (eg, decreasing the number of DTP3-unvaccinated children) as well as program process indicators focusing on cold chain, stock availability, and vaccination sessions to better describe progress on the pathway to raising immunization coverage.

**Conclusion.:**

When measuring progress toward improving immunization systems, it is helpful to use a comprehensive approach that allows for measuring multiple dimensions of the system.

## INTRODUCTION

The Polio Eradication Endgame Strategic Plan (PEESP) [1] is guided by 4 strategic objectives. The second strategic objective states that to eradicate polio, immunization systems must be strengthened in the 10 focus countries (Afghanistan, Angola, Chad, Democratic Republic of Congo, Ethiopia, India, Nigeria, Pakistan, Somalia, and South Sudan) of the Global Polio Eradication Initiative (GPEI); the 10 countries, of which 6 are in the World Health Organization (WHO) African Region, were selected because they contain significant polio assets and large numbers of unvaccinated and undervaccinated children. In carrying out this objective, the original GPEI authors of PEESP set a goal to achieve at least 10% annual increase in the third dose of diphtheria-tetanus-pertussis–containing vaccine (DTP3) coverage rates in 80% of the polio high-risk districts of the 10 focus countries from 2014 to 2018.

Shortly after development of the PEESP, the Immunization Systems Management Group (IMG) was created to monitor the second strategic objective of the PEESP implementation, and the monitoring of progress toward the 10% goal became the responsibility of the IMG Routine Immunization (IMG-RI) subgroup. This subgroup was made up of experts from multiple agencies, and they questioned the feasibility and validity of the 10% goal, particularly because historical evidence indicated that, on average, countries had increased coverage annually by 2.7% during the 1980–2009 period [2]. Additionally, considering the short timeframe (5 years) for PEESP, the subgroup members believed that a focus on efforts to strengthen immunization-system processes would be best; therefore, including process indicators alongside >1 coverage indicators would be most pertinent.

Regarding the monitoring of vaccination coverage, the subgroup did consider conducting coverage surveys; however, the cost of conducting surveys at a district level on a yearly basis was estimated in the millions when totaled across all 10 countries, and as such, was considered a very cost-ineffective approach to obtaining outcome data. The group of RI experts who comprised the IMG-RI subgroup proposed the development of a dashboard of RI indicators using existing immunization data to monitor progress toward coverage and system improvement in high-risk districts; the proposal was accepted by the IMG.

Each country in the WHO African Region has an established process of RI data collection and indicator monitoring using country-specific information systems, including Health Information Management System (HIMS), RI District Data management Module (RIM), District Vaccine Data Management Tool (DVDMT), and other local systems. The data catalogued by these systems are often referred to as country administrative data, as opposed to information obtained from vaccination coverage surveys, which generally make use of home-based records to determine vaccination status. All countries collect and submit district-level data to the WHO Regional Office on a monthly basis. These data are used to produce a regional monthly database comprised of RI coverage and numbers of children vaccinated.

Because each country in the African Region already had established processes for RI data collection, reporting, and monitoring using country-specific information systems, the IMG-RI subgroup requested that the 6 focus countries in the African Region (Angola, Chad, Democratic Republic of Congo, Ethiopia, Nigeria, and South Sudan) pilot the monitoring of a standard set of agreed-upon indicators through an RI dashboard. This paper describes the process, opportunities, and challenges for monitoring progress in RI through the establishment of a dashboard using agreed standard indicators that reflect steps in the pathway of RI strengthening with a focus on polio high-risk districts.

## METHODS

The process of developing the dashboard and the associated monitoring framework evolved with time and included the participation and input from different teams, including immunization and polio programs from the respective countries’ Ministries of Health as well as the various partners who comprised the IMG-RI subgroup (Bill and Melinda Gates Foundation; Gavi, the Vaccine Alliance; Task Force for Global Health; United Nations Children’s Fund [UNICEF]; United States Centers for Disease Control and Prevention [CDC]; and WHO).

The IMG-RI subgroup started by identifying a list of commonly collected data elements and indicators [3, 4]. This list of 15 indicators was organized around 4 performance categories: (1) the annual integrated plan for the Expanded Programme on Immunization (EPI), to assess the extent of country-level planning; (2) RI coverage improvement; (3) RI system processes, to assess how well the system is functioning and help explain the changes in coverage; and (4) RI data quality, to monitor the reliability of the data being collected. Table 1 presents the dashboard with categories, indicators, status, interpretation, data sources, reporting frequency, and reporting lag. To avoid creation of new data, the team recommended that countries use only data already being collected systematically to calculate the indicators, understanding that not all countries were collecting all exact data needed, and as such some countries would not report on some of the IMG-RI indicators.

**Table 1.** List of the Selected Indicators for Immunization Systems Management Group Routine Immunization Dashboard

Indicator definition						
	Indicator	Status	Dashboard interpretation	Data sources	Reporting frequency	Reporting lag
Annual EPI plan	Annual EPI plan includes the 5 recommended components	Plan has all 5 components	Fully met	National Immunizaiton Programme plan (Annual EPI plan)	Annually	0 months
		Plan has 3–4 components	Partially met			
		Plan has 0–2 components	Not met			
Immunization coverage improvement	% of districts that achieved ≥10% relative increase in DTP3 coverage compared with prior year	≥80%	Fully met	District-level administrative DTP3 data	Quarterly	6 months
		50–79%	Partially met			
		<50%	Not met			
	% of districts that achieved ≥10% relative decrease in the percentage of children unimmunized with DTP3 compared with prior year	≥80%	Fully met	District-level administrative DTP3 data	Quarterly	6 months
		50–79%	Partially met			
		<50%	Not met			
	National level % decrease in the number of children unimmunized with DTP3 compared with prior year	≥10%	Fully met	National level WUENIC DTP3 coverage data and national target population	Annually	8 months
		5–9%	Partially met			
		<5%	Not met			
	% of districts that achieved ≥10% relative decrease in the number of children unimmunized with DTP3 compared with prior year	≥80%	Fully met	District-level administrative DTP3 data	Quarterly	6 months
		50–79%	Partially met			
		<50%	Not met			
	% of districts that achieved a ≥10% increase in the number of children immunized with DTP3 compared with prior year	≥80%	Fully met	District-level administrative DTP3 data	Quarterly	6 months
		50–79%	Partially met			
		<50%	Not met			
Immunization processes	% of districts that achieved ≥80% scheduled fixed RI sessions conducted	≥80%	Fully met	District-level administrative data; polio staff monitoring	Quarterly	6 months
		50–79%	Partially met			
		<50%	Not met			
	% of districts that achieved ≥80% scheduled outreach RI sessions conducted	≥80%	Fully met	District-level administrative data; polio staff monitoring	Quarterly	6 months
		50–79%	Partially met			
		<50%	Not met			
	% of districts with stockouts / supply chain interruptions	<10%	Fully met	District-level administrative data; polio staff monitoring	Quarterly	6 months
		10–20%	Partially met			
		>20%	Not met			
	% districts with updated immunization microplans	≥90%	Fully met	District-level administrative data; polio staff monitoring	Annually	6 months
		70–89%	Partially met			
		<70%	Not met			
	% of districts receiving supervision from next higher level	≥80%	Fully met	District-level administrative data; polio staff monitoring	Annually	6 months
		50–79%	Partially met			
		<50%	Not met			
Immunization data quality	% of districts with complete immunization data from all health facilities for the period	≥80%	Fully met	Health facility-level administrative data	Quarterly	6 months
		50–79%	Partially met			
		<50%	Not met			
	% of districts reporting complete immunization data for the period	≥80%	Fully met	District-level administrative data	Quarterly	6 months
		50–79%	Partially met			
		<50%	Not met			
	% of districts with negative DTP1–3 dropout	<5%	Fully met	District-level administrative DTP1 & DTP3 data	Quarterly	6 months
		5–10%	Partially met			
		>10%	Not met			
	% of districts reporting >100% DTP3 coverage	<5%	Fully met	District-level administrative DTP3 data	Quarterly	6 months
		5–10%	Partially met			
		>10%	Not met			

Abbreviations: DTP, diphtheria-tetanus-pertussis–containing vaccine (numbers indicate dose); EPI, Expanded Programme on Immunization; RI, routine immunization; WUENIC, WHO/UNICEF Estimates of National Immunization Coverage.

Indicators were looked at between high-risk and non-high-risk districts. Non-high-risk districts were the ones with relatively high immunization coverage, high surveillance performance, and absence of recent circulation of polio virus, whereas the high-risk were districts with low immunity, both looking at immunization coverage and immune status of cases. Countries with support from partners developed improvement plans targeting the identified high-risk districts to increase the coverage. This is why a comparison of magnitude of changes between the 2 groups would give an idea of the impact of the intervention.

### Planning

The Annual EPI Plan was assessed in each country through measurement of 1 overall indicator, which included the following 5 components:

1. Contains SMART (specific, measurable, assignable, realistic, time-related) objectives with a harmonized calendar of supplemental immunization activities (SIAs), data quality improvement, and surveillance activities;
2. Highlights critical activities to reach all districts and communities with a focus on high-risk districts;
3. Defines clearly the roles and contributions of polio-funded assets;
4. Contains a fully costed budget with identification of funding gaps; and
5. Is endorsed by the government and the Immunization Inter-agency Coordination Committee[4].

Based on the components above, every year the IMG-RI subgroup members rated the country Annual EPI Plans after review of available operational planning materials, including Annual EPI Plans and Coverage Improvement Plans (CIPs) [5].

### Coverage Improvement

Coverage improvement was assessed in each country through 5 indicators: 1 national indicator and 4 indicators stratified by high- and non-high-risk districts. On a yearly basis, using the WHO/UNICEF Estimates of National Immunization Coverage (WUENIC), national level percentage decrease in the number of children unimmunized with DTP3 was calculated (compared with prior year). On a quarterly basis, using country administrative data and stratified by high- and non-high-risk districts, the following indicators were also calculated:

1. Percentage of districts that have achieved ≥10% relative increase in DTP3 coverage compared with prior year, which is the original PEESP systems strengthening indicator(Table 1);
2. Percentage of districts that have achieved ≥10% relative decrease in the percentage of children unimmunized with DTP3 compared with the prior year;
3. Percentage of districts that have achieved ≥10% relative decrease in the number of children unimmunized with DTP3 compared with prior year; and
4. Percentage of districts having a ≥10% increase in the number of children immunized with DTP3 compared with prior year.

### Processes Indicators

To assess the functioning of the RI system, the team selected 5 process indicators stratified by high- and non-high-risk districts, calculated using the countries’ administrative data.

The following 3 indicators were calculated on a quarterly basis:

1. Percentage of districts that have achieved ≥80% scheduled fixed RI sessions conducted;
2. Percentage of districts that have achieved ≥80% scheduled outreach RI sessions conducted[2, 7]; and
3. Percentage of districts with stock outs or supply chain interruptions. (This indicator is being collected as such in the existing information management system in countries in the African region, which why the indicate is has been phrased as it is, when one would expect it to be “percentage of districts without stock outs,” which would need additional data treatment with risk of error at country level.

The following 2 indicators were calculated on an annual basis:

4. Percentage of districts with updated immunization micro plans; and
5. Percentage of districts receiving supervision from the next higher level.

### Data Quality

To assess the quality of RI data, the team selected the following 4 indicators, stratified by high- and non-high-risk districts and calculated on a quarterly basis using the countries’ administrative data:

1. Percentage of districts with complete immunization data from all health facilities for the period [6–8];
2. Percentage of districts reporting complete immunization data for the period;
3. Percentage of districts with a negative DTP1-3 dropout (DTP1-3 dropout was calculated by subtracting the number of DTP3 doses from the number of DTP1 doses), and
4. Percentage of districts reporting >100% DTP3 coverage.

#### High-Risk Area Identification

Several methods may be used to identify polio high-risk districts in a given country [9–11]. The most common information used has included population immunity, sensitivity of disease surveillance, and insecurity. To ensure comparability of the data across countries, it was recommended that countries should use the same method and data parameters to identify the high-risk districts.

In 2011, the WHO Regional Office for Africa (AFRO) polio team developed a risk assessment tool [12] that calculates a risk level for an area based on 20 immunization coverage, surveillance, and other population and program indicators, taking into consideration the indicators’ trends during the previous 4 years (Table 2). The indicators used in the tool were identified at a global meeting on polio risk assessment held in Atlanta in July 2010 with the participation of polio staff from all WHO regions. Developed in Microsoft Excel, this user-friendly tool has been used on a quarterly basis since 2011 by >40 African Region countries to assess the risk level of their districts (Table 2). This tool was used to identify high-risk polio districts in the current project.

**Table 2.** African Region Polio Risk Assessment Indicators

No.	Variables	Value	Score
A. Surveillance			30
1	Reported WPV cases		0–7
	WPV case	Y/N	0 or 5
	If yes, orphan virus?	Y/N	0 or 2
2	Reported cVDPV or aVDPV cases		0–6
	cVDPV or aVDPV case	Y/N	0 or 4
	If yes, orphan virus?	Y/N	0 or 2
3	Meeting both AFP surveillance major indicators	Y/N	5 or 0
4	NP AFP rate (≥2)	Y/N	2 or 0
5	Proportion of AFP with 2 adequate stool specimens (≥80%)	Y/N	2 or 0
6	Proportion of AFP cases with lab results from onset to final cell culture results within 31 days (≥80%)	Y/N	2 or 0
7	Polio compatible cases		0–2
	Polio compatible cases	Y/N	0 or 1
	Cluster of polio compatible	Y/N	0 or 1
8	Proportion of AFP with inadequate stools with follow up after 60 days from onset (≥80%)	Y/N	2 or 0
9	Time since last WPV (< 6 months)	Y/N	0 or 1
10	Silent district^a^: No AFP case reported for 100000 population < 15 y per year	Y/N	0 or 1
B. Population Immunity			50
11	District administrative OPV3 routine coverage ≥90%	Y/N	17–0
12	Proportion of non polio AFP cases with ≥3 doses of OPV (≥90%)	Y/N	10–0
13	Proportion of missed children in polio SIA using end-process independent monitoring results		10; 5; 0
	<5%	Y/N	10 or 0
	5–9%	Y/N	5 or 0
	≥10%	Y/N	0
14	Timeliness of response to WPV outbreak <28 days	Y/N	8 or 0
15	At least 2 rounds of SIA after last WPV		0–5
	≥2 rounds	Y/N	5 or 0
	Not applicable	Y	5
C. Population/Program			20
16	Is the district bordering any area (district/province/country) reporting WPV	Y/N	0 or 6
17	Insecurity (UN security levels)		5; 3; 0
	Level 1–2 (low)	Y	5
	Level 3–4 (moderate/substantial)	Y	3
	Level 5–6 (high/extreme)	Y	0
18	Geographic inaccessibility (motorable roads, waterways, boats, etc)	Y/N	0 or 3
19	Population density > national average	Y/N	0 or 3
20	Special populations identified (eg, nomads, refugees, migrants, sectors, etc)? Specify	Y/N	0 or 3
		Total score	100

Abbreviations: AFP, Acute Flaccid Paralysis; cVDPV, circulating Vaccine Derived Polio Virus; NP, non Polio; OPV3, third dose of Oral Polio Vaccine; SIA, Supplementary Immunization Activities; UN, United Nations; VDPV, Vaccine Derived Polio Virus; WPV, Wild Polio Virus.

^a^If the number of children aged <15 years is less than 100000 per year, please do the following: consider a period of 2, 3, or more years for the district to report at least 1 AFP case. For example, a district with 50000 children aged < 15 years per year should only be considered as silent if no single AFP case is reported within 2 years.

### Dashboard Development and Capacity-Building Workshop

AFRO, in collaboration with the CDC, developed an Excel-based dashboard to facilitate and streamline data entry by country-level data managers. Within months of using this tool, it became apparent that countries used different methods to collect data and calculate the dashboard indicators. The IMG-RI partners responded by holding a regional workshop in June 2015, inviting data managers from AFRO regional and country offices, as well as representatives from Ministry of Health EPI programs. The goal of this workshop was to understand how the data management process used by each country varied, discuss reasons for these variations, and adopt a standardized approach to improve data comparability across all 6 African Region countries.

Among the outcomes of this workshop was a job aid designed to assist data managers with completing the dashboard data entry. Workshop participants agreed to complete the dashboard on a quarterly timeline, using the newly defined data management protocols and job aid.

This paper focuses on data collected in 2014 and 2015 because district data were not available for 2016 when the paper was drafted.

## RESULTS

The capacity building workshop and job aids development facilitated the dashboard reporting process, clarifying both indicator definitions and reporting time lines (Table 3). Despite capacity challenges and staff turnover, all 6 African countries monitored the set indicators on a quarterly basis (Tables 4 and 5). 

**Table 3.** Score Card Submission Timelines

Timing	Activity
2015 Q1 scorecard	
7 June 2015	Countries to submit 2015 Q1 scorecard to WHO AFRO IST
15 June 2015	IST to submit 2015 Q1 scorecard to AFRO
22 June 2015	AFRO to submit 2015 Q1 scorecard to CDC
2015 Q2 scorecard	
7 September 2015	Countries to submit 2015 Q2 scorecard to IST
15 September 2015	IST to submit 2015 Q2 scorecard to AFRO
22 September 2015	AFRO to submit 2015 Q2 scorecard to CDC
2015 Q3 scorecard	
7 December 2015	Countries to submit 2015 Q3 scorecard to IST
15 December 2015	IST to submit 2015 Q3 scorecard to AFRO
22 December 2015	AFRO to submit 2015 Q3 scorecard to CDC
2015 Q4 scorecard	
7 March 2016	Countries to submit 2015 Q4 scorecard to IST
15 March 2016	IST to submit 2015 Q4 scorecard to AFRO
22 March 2016	AFRO to submit 2015 Q4 scorecard to CDC

Abbreviations: AFRO, WHO Regional Office for Africa; IST, Inter-country Support Team; WHO, World Health Organization.

**Table 4.** Immunization Systems Management Group Routine Immunization Score Card 1st Quarter 2014 for Countries in the African Region

IMG Routine Immunization Monitoring DashBoard Reporting period quarter 1, 2014													
Category	Indicator	Angola		Chad		DR Congo		Ethiopia		Nigeria		South Sudan	
		HR	Non-HR	HR	Non-HR	HR	Non-HR	HR	Non-HR	HR	Non-HR	HR	Non-HR
Plan	Annual EPI plans include the 5 recommended components	100%		100%		80%		100%		100%		80%	
Outcome	% of districts that achieved ≥10% relative increase in DTP3 coverage compared with prior year	42%	47%	32%	47%	38%	30%	37%	56%	46%	41%	53%	30%
	% of districts that achieved ≥10% relative decrease in the percentage of children unimmunized with DTP3 compared with prior year	46%	71%	37%	47%	49%	44%	37%	44%	59%	50%	34%	30%
	National-level % decrease in the number of children unimmunized with DTP3 compared with prior year	21%		4%		-2%		9%		42%		–38%	
	% of districts that achieved ≥10% relative decrease in the number of children unimmunized with DTP3 compared with prior year	46%	71%	58%	59%	47%	43%	56%	67%	59%	50%	34%	33%
	% of HR districts that achieved >10% increase in the number of children immunized with DTP3 compared with prior year	46%	71%	37%	24%	44%	34%	41%	37%	52%	45%	76%	NA
Process	% of districtst that achieved ≥80% scheduled fixed RI sessions conducted	NA	NA	50%	67%	NA	NA	NA	NA	59%	76%	4%	33%
	% of districts that achieved ≥80% scheduled outreach RI sessions conducted	NA	NA	NA	NA	NA	NA	NA	NA	44%	52%	NA	NA
	% of districts with stockouts / supply chain interruptions	NA	NA	NA	NA	93%	87%	NA	NA	NA	NA	NA	NA
	% districts with updated immunization microplans	NA	NA	100%	100%	100%	100%	NA	NA	82%	82%	31%	77%
	% of districts receiving supervision from next higher level	NA	NA	53%	53%	NA	NA	NA	NA	NA	NA	20%	77%
Data quality	% of districts with complete immunization data from all health facilities for the period	NA	NA	NA	NA	NA	NA	NA	NA	39%	47%	NA	NA
	% of districts reporting complete immunization data for the period	92%	91%	100%	100%	100%	99%	NA	NA	96%	95%	43%	33%
	% of districts with negative DTP1-3 dropout	13%	11%	0%	4%	6%	5%	NA	NA	20%	12%	8%	0%
	% of districts reporting >100% DTP3 coverage	25%	40%	11%	22%	12%	21%	7%	33%	30%	33%	3%	0%

Abbreviations: DTP, diphtheria-tetanus-pertussis–containing vaccine (numbers indicate does); EPI, Expanded Programme on Immunization; HR, high-risk; IMG, Immunization Systems Management Group; RI, routine immunization.

**Table 5.** Immunization Systems Management Group Routine Immunization Score Card 4th Quarter 2015 for Countries in the African Region

IMG Routine Immunization Monitoring DashBoard Reporting period quarter 4, 2015													
Category	Indicator	Angola		Chad		DR Congo		Ethiopia		Nigeria		South Sudan	
		HR	Non-HR	HR	Non-HR	HR	Non-HR	HR	Non-HR	HR	Non-HR	HR	Non-HR
Plan	Annual EPI plans include the 5 recommended components	100%		100%		100%		100%		100%		80%	
Outcome	% of districts that achieved ≥10% relative increase in DTP3 coverage compared with prior year	37%	50%	53%	38%	49%	42%	6%	NA	59%	NA	75%	20%
	% of districts that achieved ≥10% relative decrease in the percentage of children unimmunized with DTP3 compared with prior year	49%	52%	62%	41%	36%	26%	3%	NA	67%	NA	36%	26%
	National-level % decrease in the number of children unimmunized with DTP3 compared with prior year	–2%		15%		3%		38%		12%		–16%	
	% of districts that achieved ≥10% relative decrease in the number of children unimmunized with DTP3 compared with prior year	46%	51%	62%	38%	35%	22%	3%	NA	67%	NA	23%	23%
	% of HR districts that achieved a >10% increase in the number of children immunized with DTP3 compared with prior year	43%	51%	60%	45%	53%	42%	3%	NA	61%	NA	55%	29%
Process	% of districts that achieved ≥80% scheduled fixed RI sessions conducted	NA	NA	NA	NA	49%	56%	0%	0%	85%	81%	NA	NA
	% of districts that achieved ≥80% scheduled outreach RI sessions conducted	NA	NA	NA	NA	41%	44%	0%	0%	46%	26%	NA	NA
	% of districts with stockouts / supply chain interruptions	0%	0%	0%	10%	8%	4%	0%	0%	30%	23%	NA	NA
	% districts with updated immunization microplans	NA	NA	100%	100%	100%	100%	0%	0%	77%	73%	31%	70%
	% of districts receiving supervision from next higher level	16%	56%	NA	NA	NA	NA	0%	0%	77%	68%	20%	70%
Data quality	% of districts with complete immunization data from all health facilities for the period	29%	21%	100%	100%	NA	NA	0%	0%	52%	58%	NA	NA
	% of districts reporting complete immunization data for the period	100%	99%	100%	100%	100%	100%	12%	35%	81%	78%	41%	91%
	% of districts with negative DTP1-3 dropout	23%	21%	31%	21%	15%	10%	9%	5%	18%	14%	5%	9%
	% of districts reporting >100% DTP3 coverage	17%	29%	44%	10%	0%	0%	0%	1%	60%	65%	5%	17%

Abbreviations: DTP, diphtheria-tetanus-pertussis–containing vaccine (numbers indicate dose); EPI, Expanded Programme on Immunization; HR, high-risk; IMG, Immunization Systems Management Group; RI, routine immunization.

**Figure 1.**
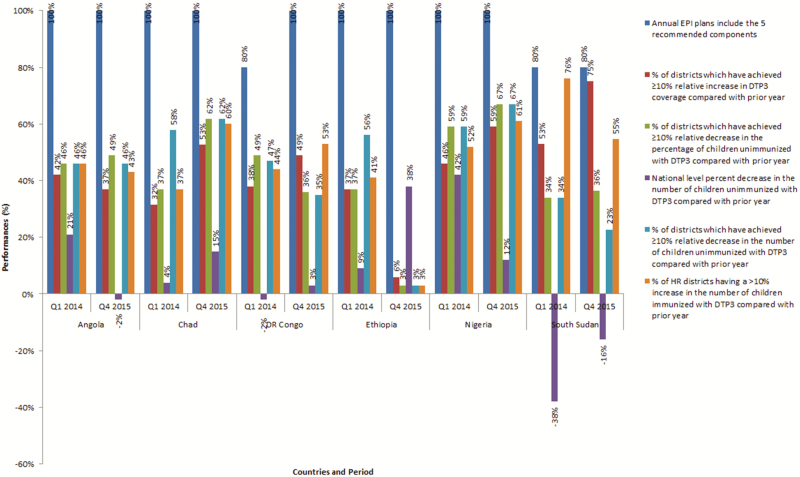
Outcome indicator performances in high-risk areas by country, fourth quarter of 2015 compared with first quarter of 2014. Abbreviations: DTP3, third dose of diphtheria-tetanus-pertussis–containing vaccine; EPI, Expanded Programme on Immunization; HR, high-risk.

Data availability and quality improved between the first quarter of 2014 and fourth quarter of 2015, especially for the process indicators driven by the impact of the country capacity strengthening (Tables 4 and 5). As indicated in Tables 4 and 5 and Figure 1, 5 countries had an annual immunization plan with the 5 required components in 2015 compared with 4 countries in 2014. The Democratic Republic of Congo and South Sudan in 2014 and South Sudan in 2015 satisfied only 4 of the 5 requirements in their plans.

**Figure 2.**
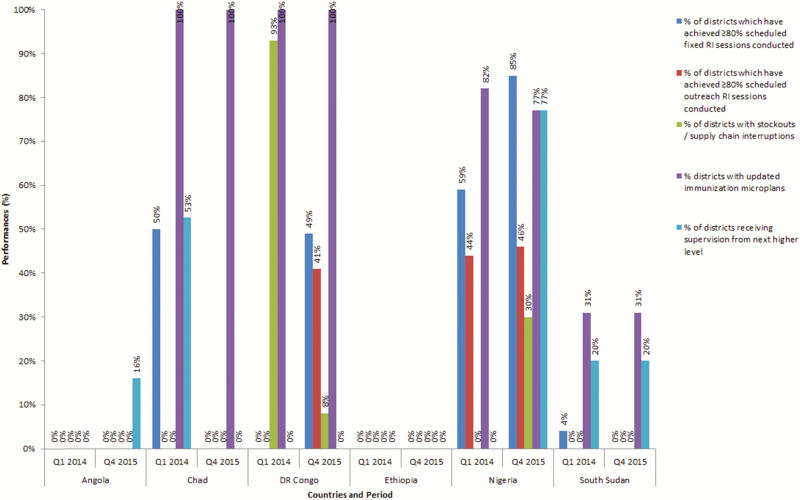
Process indicator performances in high-risk areas by country, fourth quarter of 2015 compared with first quarter of 2014. Abbreviation: RI, routine immunization.

Four countries–Chad, Democratic Republic of Congo, Ethiopia, and Nigeria–experienced national decreases of 15%, 3%, 38%, and 12%, respectively, in the number of children unimmunized with DTP3 in 2015 compared with 2014 using the latest WHO-UNICEF estimates [2] (Table 5), thought to be due in part to the improvement of the planning process in these countries and adequate implementation of the coverage improvement plan, which resulted in better vaccine availability at the operational level and better system monitoring. However, the number of children unimmunized with the third dose of DTP-containing vaccine rose by 2% and 16% in Angola and South Sudan, respectively (Table 5 and Figure 2). Chad, Ethiopia, and Nigeria have made a big jump in reduction of unimmunized children. These countries, together with the Democratic Republic of Congo constitute countries where the essential of polio asset is also located in the African Region. Polio staff is also working on RI activities in these countries now with focus on supporting the improvement plan may have had impacted these changes. Angola and South Sudan also had polio asset but did not manage to increase the coverage within the selected period.

Despite the attempt to implement the improvement plan, security challenges in South Sudan restricted access to much of the population, resulting in low coverage. South Sudan’s poor performance among the process indicators, including percentage of districts with updated immunization microplans and “percentage of districts receiving supervision from next higher level,” as well as its inability to reach 100% for planning (Table 5), helps to explain the low coverage.

Table 5 further indicates that South Sudan, despite the national decrease in vaccinating children, had the largest proportion of high-risk districts achieve a ≥10% relative increase in DTP3 coverage compared with the prior year (75%), followed by Nigeria (59%) and Chad (53%). The lowest value was found in Ethiopia, where only 6% of districs achieved the increase, whereas Democratic Republic of Congo and Angola stood at 49% and 37%, respectively.

According to the dashboard, comparison of vaccination coverage between high-risk districts and non–high-risk districts does not indicate definitive trends. This might be due to implementation of the both the reach every district (REC) and reach every community approaches in high and non-high risk districts in the African Region. These approaches are supposed to be implemented in the entire country. In the priority countries this is done with a special focus, and activities are undertaken to maintain and improve the coverage. Countries need to continue to explore innovative strategy, including correct implementation of the REC approach and use of new technology such as Geographic Information System (GIS) for microplanning, to make sure the last unreached are reached.

As shown in Tables 4 and 5 and Figure 2, 100% of districts in Chad and Democratic Republic of Congo had updated microplans for both 2014 and 2015 in both high-risk and non– high-risk districts, whereas in Nigeria there were updated microplans in >82% of both high-risk and non–high-risk districts in 2014 compared with 77% and 73% in 2015 in high-risk and non–high-risk districts, respectively.

**Figure 3.**
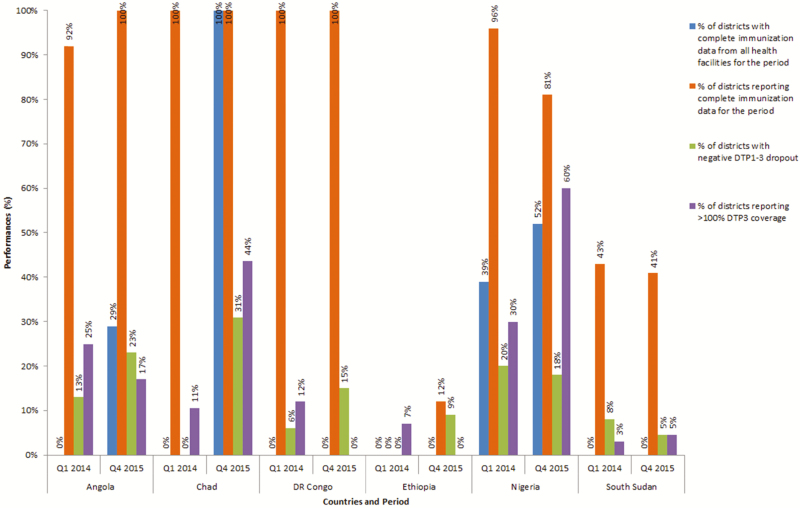
Data quality indicator performances in high-risk areas by country, fourth quarter of 2015 compared with first quarter of 2014. Abbreviation: DTP, diphtheria-tetanus-pertussis–containing vaccine (numbers indicate dose).

Tables 4 and 5 and Figure 3 further reveal that ≥90% of both high-risk and non–high-risk districts reported complete immunization data in the first quarter of 2014 and the fourth quarter of 2015 in Angola, Chad, and Democratic Republic of Congo. Nigeria achieved a district completeness of ≥90% for the first quarter of 2014 in both high-risk and non–high-risk districts; however, in the fourth quarter of 2015, it reported 81% and 78% in high-risk and non–high-risk districts, respectively. South Sudan’s district completeness in non–high-risk areas increased from 33% to 91% from the first quarter of 2014 to the fourth quarter of 2015; however, in high-risk districts, completeness remained similar at 43% and 41%, respectively (Figure 3).

## DISCUSSION

We have documented our process of creating and implementing a multidimensional monitoring tool (ie, dashboard) designed to provide information on immunization system performance. Originally developed to monitor performance of several countries during the polio eradication endgame period, the tool may also support immunization system strengthening through use to monitor progress and compare across programs. Although only a single outcome indicator goal (>10% annual increase in DTP3 coverage achieved in 80% of high-risk districts [1]) initially existed in the endgame strategy, we successfully added additional outcome indicators (eg, decreasing the number of DTP3-unvaccinated children) as well as program process indicators focusing on cold chain, stock availability, and vaccination sessions to better describe progress on the pathway to raising immunization coverage. The process of implementing the monitoring dashboard in 6 African countries provided an opportunity to improve process monitoring at a district level and highlighted the need for improvements in the availability of routinely collected process information to help inform program decision making. It appears likely that these countries will continue maintaining this process for their immunization program even beyond the IMG-RI monitoring timeframe.

Program managers may be best able to effect change when their immunization system monitoring activities include indicators for both vaccination outcomes and processes. Focusing solely on vaccination coverage will indicate too late that there is a problem; monitoring process indicators at the health facility level will be an early warning system that coverage improvements are not on track and will help program managers to determine the system components where bottlenecks exist. The system components included as process indicators (eg, working cold chain, vaccine stock management, and immunization session implementation) are contributors to coverage. Process indicators are critical to ensure that comprehensive program strategies such as RED are being implementing effectively, although routine process monitoring is not currently widely practiced in many low- and middle-income countries.

In Chad, a RED monitoring system was set up with a set of process indicators collected at health facility supervisory visits, and data from these indicators have been used in hub and district management meetings and in the annual EPI planning to analyze and address problems (Chad 2016 Annual EPI plan); coverage has improved in this country, from 48% in 2013 to 55% in 2015 (http://apps.who.int/immunization_monitoring/globalsummary/estimates?c=TCD), with immunization monitoring thought to have played a key role.

The exact formulation of process indicators will vary by country; potential indicators may include stock availability, cold chain status, and number of planned versus conducted vaccination sessions by facility and month. Compiling information on these process indicators at a higher level provides evidence on where to take action; for example, in India, polio-funded staff routinely collect data on similar indicators and discuss this information in district taskforces. Although AFRO has successfully compiled process indicator data for 5 countries (Figure 2), the use of these data for action has not yet been fully optimized. In Chad and Nigeria, process indicators are collected and analyzed locally, but the extent of systematic use in national decision-making processes can be further improved. Further efforts are required to better align the indicators to country decision-making processes and data systems. The development of this RI dashboard to monitor progress during the polio endgame period is a step in the right direction, particularly with the focus on stratifying information by high-risk and non– high-risk districts. The main perceived benefit is the systematic monitoring of the performances within and across countries using standard parameters and set periodicity.

Important limitations include the quality of the administrative data and staff turnover affecting the capacity of the country to maintain the system. To address coverage based on administrative data, we used WHO/UNICEF estimates for national coverage and supported countries to conduct regular data quality assessments and self-assessment to identify gaps and implement corrective actions or data quality improvement plans. These latter activities were reported to be useful and hopefully will continue. Additionally, countries should increase the number of staff who are trained to use the dashboard to ensure its sustainability and benefits.

In comparison, malaria programs currently use a scorecard under the African Leaders Malaria Alliance (ALMA) initiative (http://alma2030.org/) that can provide lessons to immunization programs on the usefulness of visual aids on program performance. AFRO and the UNICEF regional offices in Africa and other immunization partners are exploring these lessons and their use in advocacy among African heads of state, as a follow-up of the Ministerial Conference on Immunization in February 2016 (http://immunizationinafrica2016.org/).

The RI system is complex and should be monitored using a multidimensional approach because this allows for program staff to identify bottlenecks that may be inhibiting program outcomes. Additionally, outcome goals should be realistic and reflect the input of those most knowledgeable about the potential achievements possible during short timeframes. Dashboard monitoring has the potential for providing many benefits to those countries that continue to use it beyond polio eradication but will only prove useful if it is included in local and national decision-making processes and not only used at a global level.

## Notes

***Financial support.*** No specific funding was provided but resources from the Bill and Melinda Gates Foundation, the Centers for Disease Control and Prevention, GAVI, UNICEF, and WHO supported this work.

***Supplement sponsorship.*** This work is part of a supplement coordinated by the Task Force for Global Health with funding provided by The Bill and Melinda Gates Foundation and the Centers for Disease Control and Prevention.

***Potential conflicts of interest.*** All authors: No reported conflicts.
